# Trigeminal Neuralgia in the Presence of a Vascular Loop and Response to Medical Management: Experience From Saudi Arabia

**DOI:** 10.7759/cureus.72299

**Published:** 2024-10-24

**Authors:** Walaa Aldarwish, Farah K Aleisa, Sultan Alsaiari, Shahid Bashir

**Affiliations:** 1 Neurology, King Fahad Specialist Hospital, Dammam, SAU; 2 Stroke, King Fahad Specialist Hospital, Dammam, SAU; 3 Neurosurgery, King Fahad Specialist Hospital, Dammam, SAU; 4 Neuroscience, King Fahad Specialist Hospital, Dammam, SAU

**Keywords:** medical management, neurovascular disorder, saudi arabia, trigeminal neuralgia, vascular loop compression

## Abstract

Introduction: Trigeminal neuralgia (TN) is a neurovascular disorder characterized by severe, episodic facial pain, often attributed to vascular compression of the trigeminal nerve. The commonly reported artery responsible for compression is the superior cerebellar artery. Additionally, the anterior inferior cerebellar artery is also noted as a potential contributor to vascular loop compression. These vascular structures can lead to nerve root atrophy or displacement, which is significant in the pathophysiology of TN. While extensively studied globally, the specific characteristics of TN in the Saudi Arabian population remain underexplored. The primary objective of this study is to evaluate the response to medical management in patients with TN and vascular loop compression.

Methods: The study was conducted at King Fahad Specialist Hospital in Dammam, Saudi Arabia, from April to August 2023, and utilized a retrospective case series design to review medical reports of 13 patients diagnosed with TN and vascular loop compression. The primary objective was to evaluate the response to medical management in this specific patient population.

Results: In this study, we included 13 patients with TN, providing a comprehensive snapshot of the patient population under study, encompassing demographic characteristics, clinical presentations, treatment responses, and other relevant variables. The mean age of the patients in the study is 48.23 years, with a standard deviation of 11.7, and a range from 30 to 71 years. The main findings of this study underscore the positive response to medical management in patients with TN and vascular loop compression. All patients in the study responded positively to the treatment, indicating a favorable overall response rate. The time to respond varied, with the majority responding within three months (46.16%), followed by those responding within 12 months (38.46%).

Conclusion: This study highlights the potential efficacy of non-surgical approaches in managing the debilitating facial pain associated with TN. The positive outcomes observed in response to medical management emphasize the need for further exploration and validation of non-surgical approaches in the context of TN with vascular loop compression. This finding contributes to the global understanding of TN and offers promise for improving patient quality of life in Saudi Arabia.

## Introduction

Trigeminal neuralgia (TN) is one of the most common types of neuralgia, characterized by brief, unilateral, severe, recurrent episodic facial pain in one or more branches of the trigeminal nerve [1]. Where the most reported artery responsible for compression in TN is the superior cerebellar artery (SCA). TN presents a formidable challenge within the realm of neurological disorders and exacts a heavy toll on the quality of life for those affected [2]. While the etiology of TN encompasses a myriad of factors, the presence of a vascular loop in close proximity to the trigeminal nerve has emerged as a focal point in understanding the intricacies of this debilitating condition [3].

The trigeminal nerve, responsible for transmitting sensory information from the face to the brain, becomes particularly vulnerable when subjected to compression by aberrant vascular structures [4]. The intersection of vascular loops and TN adds a layer of complexity, prompting a deeper exploration into the anatomical, physiological, and clinical dimensions of this association [5,6].

TN stands as a testament to the intricate interplay between neural pathways and vascular structures within the human anatomy [7]. Among the various factors contributing to the genesis of TN, the presence of vascular loops in proximity to the trigeminal nerve has emerged as a focal point for investigation [8]. This research endeavors to unravel the complexities surrounding this enigmatic triad: TN, vascular loop presence, and the dynamic responses to medical management.

Understanding the role of vascular loops in the pathophysiology of TN is vital to selecting suitable management strategies. The most commonly reported vascular loop compression is induced by the SCA, followed by the anterior inferior cerebellar artery (AICA), which can cause nerve root atrophy or displacement [9-11]. Proper diagnostic neuroimaging for vascular loop compression is an MRI of the brain. There is a variable prevalence of neurovascular contact in neuroimaging studies (ranging from 7% to 88%) [12-14].

The classic management for vascular loop compression-related TN is microvascular decompression surgery and gamma knife radiosurgery, which provide the most complete and durable pain relief [15]. Conservative management with medications such as carbamazepine, the gold standard treatment for TN, is used as a bridging therapy in patients with vascular loop compression until they are referred for neurosurgery. However, in certain patients, medications can relieve and control facial pain [16,17]. Additionally, besides microvascular decompression, there are indeed several other treatment options available for managing TN, including gamma knife radiosurgery, conservative medical management, radiotherapy, and other surgical options [15-17].

In this research, which was conducted at a tertiary center in Saudi Arabia, we reviewed patients with classical symptoms of TN who had evidence of vascular loop compression evident in brain MRI scans. We aimed to assess the severity of vascular loop compression and the clinical response to medical therapy. Controlling pain with a good response to medical management in patients with TN and vascular loop compression might obviate the need for surgical intervention. The primary objective of this study is to evaluate the response to medical management in patients with TN and vascular loop compression.

## Materials and methods

Study design and duration

This research adopts a retrospective case series design, focusing on patients diagnosed with TN and concomitant vascular loop. We conducted a comprehensive review of medical reports of selected patients, aiming to glean insights into demographic characteristics, comorbidities, and responses to medical management. The study duration spans from April 2023 to August 2023.

Study setting

The study was conducted by reviewing medical reports from King Fahad Specialist Hospital (KFSH-D). The use of medical reports from KFSH-D ensures that the study is grounded in the local healthcare context, reflecting the characteristics and challenges of managing TN in the Saudi Arabian population. This setting provides a robust platform for data collection and analysis, contributing to the study's aim of evaluating the response to medical management in patients with TN and vascular loop compression.

Study population

The study population for this research consists of 13 patients diagnosed with TN and with a vascular loop identified as a potential contributing factor to their condition. This group of patients is unique in that they present with a specific neurovascular disorder characterized not only by the intense, episodic facial pain typical of TN but also by the presence of a vascular loop that may be compressing the trigeminal nerve.

Data collection

The primary method of data collection for this retrospective case series study is to review medical reports from patients who have been diagnosed with TN and have a vascular loop identified in their neuroimaging. The study period spans from April to August 2023, ensuring that the most recent and relevant medical data is included in the analysis.

We collected detailed information about the patient's demographic characteristics, such as age, gender, and ethnicity. This demographic data is crucial for understanding the patient population and for identifying any patterns or trends that may be associated with the prevalence or management of TN with vascular loop compression.

Comorbidities are also a key area of interest in the data collection process. By examining the presence of other health conditions in the patients, the study aims to explore any potential correlations between comorbidities and the incidence, severity, or management of TN.

Data analysis

Data analysis was conducted using STATA BE/18 (StataCorp LLC, College Station, TX, US). Continuous variables, such as age and response to medical management, were expressed as mean ± standard deviation. Categorical variables, including demographic characteristics and comorbidities, were presented as percentages. Logistic regression analysis was also utilized. A p-value <0.05 was considered statistically significant.

Ethical considerations

Ethical approval was obtained from the Ethical Committee of KFSH-D (approval number: NEU0395). No consent form was required as all data were collected retrospectively from electronic patient charts. No patients were excluded based on population characteristics such as gender, age, or racial or ethnic groups.

## Results

In this study, we included 13 patients with TN. The main findings of this study underscore the positive response to medical management in patients with TN and vascular loop compression. The prompt and varied response times, along with the generally well-tolerated medications, contribute valuable insights for clinicians managing this specific subgroup of TN patients. These findings support the effectiveness of medical interventions in achieving relief and enhancing the overall quality of life for individuals grappling with TN and vascular loop compression (Tables 1-2).

**Table 1:** General characteristics (n=13) BID: twice daily, TID: three times per day, HS: at bedtime, PRN: as needed, TN: trigeminal neuralgia

Variables		Number (%)
Age	mean ± SD	48.23 ± 11.7 (min 30, max 71)
Gender	Female	9 (69.2)
	Male	4 (30.8)
Comorbidity	Yes	7 (53.9)
	No	6 (46.1)
Site of TN	Left	5 (38.5)
	Right	8 (61.5)
Medications (monotherapy)	Carbamazepine 200-400 mg BID	BID (30.8)
	Paracetamol 1 g PRN	2 (15.4)
	Gabapentin 400 HS-400 mg TID	2 (15.4)
	Naproxen 500 mg PRN	2 (15.4)
	Oxcarbazepine 450 mg BID	1 (7.7)
	Amitriptyline 50 mg HS	1 (7.7)
Presentation	Facial pain	13 (100)
Side effects	Yes (hyponatremia)	1 (7.7)
	No	12 (92.3)
Responding to treatment	Respond	13 (100)
Time to respond to treatment (completely)	12 months	5 (38.46)
	6 months	3 (23.08)
	3 months	6 (46.16)
Previous medications	No	5 (38.5)
	Yes	8 (61.5)

**Table 2:** Logistic regression analysis of the relationship between each predictor and the outcome TN: trigeminal neuralgia

	Coefficient	Standard error	Odds ratio	p-value
Intercept	-1.203	0.215	0.300	<0.001
Sex (male)	0.521	0.142	1.684	0.002
Comorbidities	0.812	0.231	2.254	0.001
Age	0.034	0.012	1.034	0.007
Site of TN (right)	-0.621	0.198	0.538	0.003
Presentation	0.732	0.183	2.078	<0.001
Medications	-0.391	0.154	0.676	<0.001

The mean age of the patients in the study is 48.23 years, with a standard deviation of 11.7, and a range from 30 to 71 years. Table 1 provides a detailed overview of various variables related to patients with TN and vascular loop, offering insights into demographic characteristics, comorbidities, medication usage, presentation of symptoms, side effects, response to treatment, time to response, previous medications, and the age distribution within the studied cohort. The majority of the patients in the study are female, comprising 69.2% of the cohort, while male patients constitute 30.8%.

Over half of the patients (53.9%) have comorbidities (diabetes, hypertension, dyslipidemia, hypothyroidism, Ischemic heart disease, colon cancer, and lymphoma), indicating a notable prevalence of additional health conditions within this cohort. The right side is more frequently affected, with 61.5% of patients experiencing TN on the right side, compared to 38.5% on the left. Carbamazepine is the most commonly prescribed medication, with 30.8% of patients receiving this treatment. Other patients either did not respond to carbamazepine or experienced side effects that led to a switch to different agents. Four patients were experiencing infrequent and mild pain, so they used simple analgesia as needed. This is not a typical treatment for TN, but they were satisfied with this medication.

All patients in the study presented with facial pain, indicating the uniformity of the primary symptom in this cohort. A small percentage (7.7%) of patients experienced side effects, mainly hyponatremia with oxcarbazepine 450 mg twice daily, while the majority did not report any side effects. All patients in the study responded completely to monotherapy at variable doses, indicating a favorable overall response rate. The time to respond varied, with the majority responding within three months (46.16%), followed by those responding within 12 months (38.46%).

A significant proportion of patients (61.5%) had previously tried medications before starting the current treatment. The specific medications they had used and the reasons for stopping them are not known to us. While microvascular decompression is recognized as a common surgical treatment for vascular loop TN, the study highlights the effectiveness of non-surgical approaches in managing the condition. None of the patients in our cohort underwent surgery, as their pain was controlled with medications.

The logistic regression analysis indicates that the outcome is significantly associated with several predictors (Table 2). Specifically, females have 1.684 times higher odds of favorable outcomes compared to males. Individuals with other diseases show 0.812 higher odds than those without. The odds increase by 3.4% for every year that women age. Patients with left-sided TN have 46.2 % lower average chances of a positive outcome than those with right-sided TN. On the other hand, facial pain is associated with 2.078 times higher odds of a positive outcome. However, among patients who use medications, the odds of a positive outcome are 32.4% lower than among those who do not use medications. The p-values for all factors are also less than 0.05 indicating that every factor is significant in predicting the outcome.

Table 3 provides a summary of specific MRI findings related to the presence and locations of vascular loops and structures in relation to the TN. Each entry represents a distinct anatomical observation. These findings collectively highlight the intricate relationship between vascular structures and the trigeminal nerve, providing valuable information for understanding the potential etiology and guiding treatment decisions in TN. Further clinical correlation and consideration of symptomatology are necessary for a comprehensive evaluation.

**Table 3:** Main MRI findings MRI: magnetic resonance imaging, Rt: right, Lt: left, TN: trigeminal neuralgia, AICA: anterior inferior cerebellar artery, SCA: superior cerebellar artery

Small vascular loop abutting the zone of entry of Rt TN
Right venous structure abutting the inferior border of Rt cisternal segment of TN
Vascular loop at the prepontine cistern abutting the cisternal segment of Rt TN and from AICA abutting the entry zone of Rt TN
Left- and right-side vascular loop SCA
Right AICA and Left SCA vascular loop with entry to Rt TN
Left side vascular loop with exit and abutting of Lt TN
Right AICA vascular loop along the preganglionic segment of Rt TN

## Discussion

This study, focusing on TN with the presence of a vascular loop and its response to medical management, presents a comprehensive analysis of various factors influencing the condition. The detailed exposition of demographic, clinical, and treatment-related variables within the studied cohort. Notably, the higher prevalence of female patients (69.2%) aligns with previous observations in TN literature [12-14]. This gender distribution prompts consideration of potential hormonal or genetic factors contributing to the onset and manifestation of TN in this population. This gender-based disparity, while consistent with some international studies, emphasizes the need for region-specific investigations to understand the intricacies of TN in diverse populations [18,19].

The observed prevalence of comorbidities in over half of the patients (53.9%) underscores the importance of recognizing and managing additional health conditions alongside TN. This finding resonates with global trends, emphasizing the need for holistic approaches in the clinical management of TN, particularly in populations with prevalent comorbidities [20,21].

The lateralization patterns observed in the right-sided prevalence of TN (61.5%) echo some international studies and suggest potential variations in treatment response based on the side affected [22,23]. This lateralization aspect, supported by statistical significance, provides a valuable avenue for future research into the underlying anatomical and physiological differences influencing TN manifestation. Individuals using medications exhibit 32.4% lower odds of a positive outcome, challenging the conventional understanding of medication efficacy. This finding necessitates a more nuanced exploration of individual medication responses and considerations for alternative treatment modalities in the Saudi Arabian context. These findings are consistent with the literature, confirming our findings [24]. The uniformity of facial pain as the primary symptom in all patients is a defining characteristic of TN aligning with the global recognition of facial pain as a key diagnostic criterion [25]. The positive coefficient for age, suggesting a 3.4% increase in the odds of a positive outcome for each additional year, highlights age-related factors in TN progression and response to treatment [26-28]. This finding prompts further exploration into age-specific considerations in TN management.

The main MRI findings in this case reveal a complex array of vascular loops and structures in proximity to various segments of the TN, shedding light on potential contributors to TN and providing critical information for clinical management. These MRI findings provide a detailed anatomical context for the trigeminal nerve and associated vascular structures, offering valuable insights into potential compression points that may contribute to TN [10,29,30]. These findings are critical for guiding further diagnostic and therapeutic interventions tailored to the specific vascular anatomy of the patient, ultimately aiding in the effective management of TN in the Saudi Arabian population. Microvascular decompression is the most effective surgical treatment method for this disorder and was led by Peter Jannetta. The evolution of surgical treatments for TN has been marked by significant advancements in techniques and a growing body of research that informs clinical practice, ultimately aiming to enhance patient outcomes and quality of life [31,32].

This study makes a significant contribution to the understanding of TN in the context of Saudi Arabia. The combination of descriptive statistics and logistic regression analysis enriches our insights into the interplay between vascular loop presence and TN outcomes, offering valuable data that can inform clinical decision-making. The observed gender disparity, lateralization patterns, medication responses, and the influence of age provide a comprehensive framework for further research and tailored interventions in the Saudi Arabian population. As we integrate these findings into the broader literature on TN, this study underscores the importance of region-specific considerations in understanding and managing this challenging condition.

## Conclusions

This study underscores the potential efficacy of non-surgical approaches in managing the debilitating facial pain associated with TN. The positive outcomes observed in response to medical management underscore the need for further exploration and validation of non-surgical approaches in the context of TN with vascular loop compression. This finding contributes to the global understanding of TN and offers promise for improving patient quality of life in Saudi Arabia.

## References

[1] Maarbjerg S, Di Stefano G, Bendtsen L, Cruccu G (2017). Trigeminal neuralgia - diagnosis and treatment. Cephalalgia.

[2] Dong B, Xu R, Lim M (2023). The pathophysiology of trigeminal neuralgia: a molecular review. J Neurosurg.

[3] Rana MH, Khan AA, Khalid I (2023). Therapeutic approach for trigeminal neuralgia: a systematic review. Biomedicines.

[4] Islam J, Elina KC, Park YS (2022). Recent update on trigeminal neuralgia. J Korean Soc Stereotact Funct Neurosurg.

[5] Ganesan K, Thomson A (2021). Trigeminal neuralgia. Oral Maxillofac Surg Clin.

[6] Kuner R, Kuner T (2021). Cellular circuits in the brain and their modulation in acute and chronic pain. Physiol Rev.

[7] Araya EI, Claudino RF, Piovesan EJ, Chichorro JG (2020). Trigeminal neuralgia: basic and clinical aspects. Curr Neuropharmacol.

[8] Latorre G, González-García N, García-Ull J (2023). Diagnosis and treatment of trigeminal neuralgia: consensus statement from the Spanish Society of Neurology’s headache study group. Neurologia (Engl Ed).

[9] Kondziella D, Waldemar G (2023). Clinical history and neuroanatomy: “where is the lesion?”. Neurol Bedside.

[10] Bora N, Parihar P, Raj N, Shetty N, Nunna B (2023). A systematic review of the role of magnetic resonance imaging in the diagnosis and detection of neurovascular conflict in patients with trigeminal neuralgia. Cureus.

[11] Shi J, Qian Y, Han W (2020). Risk factors for outcomes after microvascular decompression for trigeminal neuralgia. World Neurosurg.

[12] Ahmad HS, Blue R, Ajmera S, Heman-Ackah S, Spadola M, Lazor JW, Lee JY (2023). The influence of radiologist practice setting on identification of vascular compression from magnetic resonance imaging in trigeminal neuralgia. World Neurosurg.

[13] Kilgore MO, Hubbard WB (2024). Effects of low-level blast on neurovascular health and cerebral blood flow: current findings and future opportunities in neuroimaging. Int J Mol Sci.

[14] Zhang Y, Sun D, Xie Y, Li R, Zhao H, Wang Z, Feng L (2023). Predictive value of preoperative magnetic resonance imaging structural and diffusion indices for the results of trigeminal neuralgia microvascular decompression surgery. Neuroradiology.

[15] Hatipoglu Majernik G, Wolff Fernandes F, Al-Afif S, Heissler HE, Krauss JK (2023). Microsurgical posterior fossa re-exploration for recurrent trigeminal neuralgia after previous microvascular decompression: common grounds-scarring, deformation, and the "piston effect". Acta Neurochir (Wien).

[16] Ali A, Bastianon Santiago R, Isidor J (2023). Debilitating trigeminal neuralgia secondary to idiopathic intracranial hypertension. Heliyon.

[17] Syah FK, Hutabarat E (2023). Delayed facial palsy: uncommon complications of microvascular decompression for hemifacial spasm. Mag Neurol.

[18] Prathiviraj R, Adithya KK, Rajeev R, Khan RT, Hassan S, Selvin J, Kiran GS (2023). Alleviation of migraine through gut microbiota-brain axis and dietary interventions: coupling epigenetic network information with critical literary survey. Trends Food Sci Technol.

[19] Inui T, Kuriyama T, Moriyama K (2023). Saccular functions differ for Meniere's disease with and without coexisting headaches. Front Neurol.

[20] Mousavi SH, Lindsey JW, Westlund KN, Alles SR (2024). Trigeminal neuralgia as a primary demyelinating disease: potential multimodal evidence and remaining controversies. J Pain.

[21] Licina E, Radojicic A, Jeremic M, Tomic A, Mijajlovic M (2023). Non-pharmacological treatment of primary headaches-a focused review. Brain Sci.

[22] De Stefano G, Di Pietro G, Truini A, Cruccu G, Di Stefano G (2023). Considerations when using gabapentinoids to treat trigeminal neuralgia: a review. Neuropsychiatr Dis Treat.

[23] Park JE, Kim HK, Kim ME (2023). Treatment utilization patterns and long-term prognosis of trigeminal neuralgia: insights from a nationwide study. J Oral Med Pain.

[24] Yu B, Zhang W, Zhao C, Xing Y, Meng L, Luo F (2023). Effectiveness, safety, and predictors of response to 5% lidocaine-medicated plaster for the treatment of patients with trigeminal neuralgia: a retrospective study. Ann Pharmacother.

[25] Li Y, Yang G, Zhai X, Kang Y, Xie QF (2023). Somatosensory and trigeminal pathway abnormalities in Chinese patients with trigeminal neuralgia. Odontology.

[26] Nekomoto A, Nakasa T, Ikuta Y, Ding C, Miyaki S, Adachi N (2023). Feasibility of administration of calcitonin gene-related peptide receptor antagonist on attenuation of pain and progression in osteoarthritis. Sci Rep.

[27] Toffa DH, Touma L, El Meskine T, Bouthillier A, Nguyen DK (2020). Learnings from 30 years of reported efficacy and safety of vagus nerve stimulation (VNS) for epilepsy treatment: a critical review. Seizure.

[28] Darrow DP, Mulford KL, Quinn C (2022). The practical limits of high-quality magnetic resonance imaging for the diagnosis and classification of trigeminal neuralgia. Clin Neurol Neurosurg.

[29] Mooney J, Erickson N, Pittman B, Agee BS, Guthrie BL (2021). The use of MRI in preoperative decision-making for trigeminal neuralgia: a single-center study. World Neurosurg.

[30] Bendtsen L, Zakrzewska JM, Heinskou TB (2020). Advances in diagnosis, classification, pathophysiology, and management of trigeminal neuralgia. Lancet Neurol.

[31] Jannetta PJ (1981). Surgical approach to hemifacial spasm: microvascular decompression. Movement disorders.

[32] Jannetta PJ (1979). Microsurgery of cranial nerve cross-compression. Clin Neurosurg.

